# Case autoimmune pancreatitis in children

**DOI:** 10.1186/1546-0096-12-S1-P287

**Published:** 2014-09-17

**Authors:** Alicia G Cazares Camacho, Maria Maldonado, Enrique Faugier, Maria de Lourdes Cabrera

**Affiliations:** 1 Reumatologia pediatrica, Hospital Infantil de Mexico Federico Gomez, Mexico City, Mexico; 2 Patologia, Hospital Infantil de Mexico Federico Gomez, Mexico City, Mexico

## Introduction

Autoimmune pancreatitis represents 2 % of chronic pancreatitis , most often in adult male presentation associated with IgG4. We present a case of a patient 6 years old, with a dependent pancreatic and bile duct obstruction abdominal mass, which is interesting because this presentation in children is more common with autoimmune pancreatitis that malignancies.

## Objectives

Show an exceptional case in pediatrics rheumatology.

## Methods

Presentation of case.

## Results

Female 6 years old, previously healthy, 3-year evolution gastric recurrent vomiting, abdominal pain intermittently mesogastrio , adding hiporexia, jaundice and increased waist circumference, with palpable mass in the right upper quadrant. Cholestatic syndrome, 857 Lipase, amylase 137 Immunoglobulin IgG subclass 4: 23mg/dL (5-6 years: 1-121). CT: dilatation of intra and extra hepatic bile duct. Level space-occupying hepatic hilum extending to pancreatic head and esophagogastric junction mass. Gonadotropin and alpha-fetoprotein: negatives. Cholecystectomy, incisional biopsy of tumor, liver biopsy was performed. He reported: lymphoplasmacytic sclerosing pancreatitis (chronic autoimmune pancreatitis).

## Conclusion

This is a rare autoimmune disorder that resembles a pancreatic neoplasm with biliary obstruction, occurring primarily in adults, making it an exceptional case in pediatrics. As part of an IgG4 -associated systemic disease, serum level may be normal in up to 40 %, being positive in pancreatic tissue.

## Disclosure of interest

None declared.

